# Sitagliptin phosphate ameliorates chronic inflammation in diabetes mellitus via modulating macrophage polarization

**DOI:** 10.3389/fendo.2025.1544684

**Published:** 2025-04-07

**Authors:** Xiaoxia Hu, Yalong Li, Xinyue Liu

**Affiliations:** The Second Hospital & Clinical Medical School, Lanzhou University, Lanzhou, Gansu, China

**Keywords:** diabetes, Sitagliptin phosphate, inflammation, macrophages, PPAR-γ

## Abstract

**Aim:**

To investigate the effect and mechanism of Sitagliptin phosphate on inflammation and macrophage polarization in a mouse model of type 2 diabetes.

**Methods:**

*In vitro*, Raw264.7 cells were cultured with a high concentration of glucose (HG) and sitagliptin phosphate (SIG). The levels of inflammatory factors and the regulation of macrophage polarization were investigated, and the differentially expressed genes between HG and HG+SIG intervention were analyzed and enriched through transcriptomics. *In vivo*, C57BL/6J male mice were treated with HFD+STZ to establish a type 2 diabetes mouse model were investigated the effects of regulation of macrophage polarization in the pancreas and visceral adipose tissue.

**Results:**

In vitro cell experiments and transcriptomics showed that Sitagliptin phosphate decreased the secretion of inflammatory factors IL-6 and TNF-α induced by high-glucose, and increased secretion of anti-inflammatory factor IL-10 by enhancing macrophage polarization. In vivo, the body weight and abdominal visceral fat weight, the ratio of visceral fat weight to body weight and fasting blood glucose were significantly increased in the DM group compared with the Control (*P*<0.05), Sitagliptin phosphate treatments reversed the changes in the DM group. Moreover, histological analysis showed that compared with the Control group, the size of visceral adipocytes, hepatocyte lipid deposition and the ratio of M1/M2 macrophage were higher in the DM group, which were reversed by Sitagliptin phosphate treatments (*P*<0.05), insulin treatments did not have a significant effect (*P*>0.05). Mechanistically, Western blot showed that compared with the normal group, HG upregulated the expression of mTORc1 protein, P-65 phosphorylation and P-65 protein expression in Raw264.7 cells (*P*<0.05), downregulated the expression of IKKβ (*P*<0.05) and PPAR-γ proteins (*P*<0.05), Sitagliptin phosphate and insulin treatments rescued these changes.

**Conclusion:**

These results indicated that Sitagliptin phosphate reduced high glucose-induced inflammation by improving the imbalance of macrophage polarization via modulating the mTORc1/ PPAR-γ/NF-κB *in vitro* and *in vivo*.

## Introduction

The global prevalence of diabetes has been on the rise in recent years. This has imposed a significant burden on the patients and society. Diabetes is a metabolic disease which is characterized by elevated high blood sugar with the potential to cause other complications, including hyperlipidemia, fatty liver, et al. Reports by the International Diabetes Federation (IDF) released in December 2021 showed an estimated 537 million individuals between the aged of 20 and 79, affected by diabetes worldwide. Notably, the 141 million patients are from China, making it the most affected country. The number of diabetes patients in China is expected to reach 164 million by 2030. This number is expected to reach 783 million by 2045 globally, with 174 million cases attributed to China (1). Multiple glucose-lowering drugs have been developed to treat diabetes via different mechanisms, however, their efficacy in achieving sufficient blood sugar control is sup-optimal, and the treatment compliance rate in China is still low. Zhong et al. (2) reported that the diabetes control rate for adults with diabetes in China was 64.1% as evidenced by Glycosylated Hemoglobin (GHbA_1_C) tests. Patients with diabetes who have the same blood sugar levels may demonstrate varying degrees of glycemic control despite receiving identical treatments. Additionally, achieving optimal blood sugar levels alone does not prevent the long-term complications associated with diabetes, underscoring the necessity for new therapeutic targets for effective treatment.

Recent studies have classified diabetes as a chronic systemic inflammatory metabolic disease. The recruitment, accumulation, and activation polarization of macrophages in metabolic tissues such as adipose tissue and the pancreas play a crucial role in the onset of chronic low-grade inflammation (3). Chronic inflammation is the primary factors driving the onset and progression of type 2 diabetes mellitus (T2DM) (4) and its associated vascular complications, such as non-alcoholic fatty liver disease (NAFLD), retinopathy, nephropathy, and cardiovascular disease (5, 6). Consequently, research on macrophages has intensified in recent years. Dipeptidyl Peptidase-4 (DPP-4) inhibitors lower blood sugar levels by enhancing insulin secretion, facilitating the proliferation of pancreatic β-cells, delaying gastric emptying, and reducing appetite (7).Beyond their role in glucose homeostasis, studies on the pleiotropic effects of DPP-4 inhibitors have demonstrated that DPP-4 contributes to adipose tissue inflammation in obese individuals by modulating macrophage polarization imbalance (8), DPP-4 inhibitors decreases lipid deposition in the liver of mice fed with high-fat diet (9–11) and reduces the total fat content in patients with T2DM (12, 13), and regulating the Peroxisome proliferator-activated receptor-γ(PPAR-γ) functions (14). Sitagliptin phosphate, a DPP-4 inhibitor, is increasingly being investigated for its potential to alleviate chronic inflammation associated with diabetes and the mechanisms involved.

In this study, we investigated the effect of Sitagliptin phosphate on inflammation and macrophage polarization in a mouse model of T2DM, as well as explored the underlying mechanisms.

## Materials and methods

### Animals

Animal care and experimental procedures were approved by the Animal Ethics Committee of the Second Hospital of Lanzhou University. C57BL/6J male mice (6-8 weeks old, n=40) were purchased from the Lanzhou Veterinary Research Institute of the Chinese Academy of Agricultural Sciences. After 1 week of adaptive feeding, the mice were randomly assigned to receive a normal diet (4.3% (w/w) fat content, 10% kcal) (NC group, n = 10) or a high-fat diet (35% (w/w) fat content, 60% kcal) (HFD group, n = 30), and body weight and fasting blood glucose were measured every 2 weeks for 4 weeks. After the fourth week, the mice in the HFD group were intraperitoneally injected with 50 mg/kg of streptozotocin (STZ, Sigma, dissolved in 0.05M, pH 4.5 sterile sodium citrate) for 3 consecutive days. Blood samples were collected from the tail vein on the 3rd, 5th, and 7th days after intraperitoneal injection for the measurement of fasting blood glucose and random blood glucose. Mice were classified as hyperglycemia if their fasting blood glucose levels exceeded 200 mg/dL (11.1 mmol/L) or if their random blood glucose levels were greater than 300 mg/dL (16.7 mmol/L). The hyperglycemia mice were continuously fed with a high-fat diet for 1 week, and the fasting blood glucose was retested. If the fasting blood glucose was still higher than 200 mg/dl (11.1 mmol/L), the T2DM animal model was deemed to be successful es. The successfully modeled T2DM mice were randomly divided into T2DM group (DM group, n=10, pure water 10 mg/kg/d, gavage, once a day), a Sitagliptin phosphate group (SIG group, n=10, Sitagliptin phosphate, Sigma, 10 mg/kg/d, gavage, once a day), and an insulin group (Insulin group, n=10, insulin Degludec, Novo Nordisk, 1-2 U IH, once a day), for 8 weeks. At the end of the experiment, the mice were subjected to a 12-hour fasting period from 8 PM to 8 AM, after which body weight and fasting blood glucose levels were recorded. Following intraperitoneal injection of 1% chloral hydrate for anesthesia, blood samples were obtained from the inferior vena cava. Subsequently, visceral fat, liver, pancreas, and other tissues were harvested from each mouse for further analysis.

### Cell culture and experiments

Raw264.7 cell line was purchased from China Center for Type Culture Collection.Raw 264.7 cells were routinely subcultured in complete DMEM medium containing 10% FBS, 5% CO_2_, and a cell culture incubator at 37°C. After the cells reached a confluence of 80%~90%, the cells were subcultured at a ratio of 1:3. After 3~5 routine subcultures, healthy cells of the same generation in the logarithmic growth phase were taken and inoculated in a 6-well cell culture plate at 2.0×10^6^/ml for drug experiments. Based on the results of the preliminary experiments, the high-glucose group was prepared with a concentration of 35 mmol/L and a stimulation of 6 hours. The cells were divided into four groups as follows: (1) NC, (2) HG, (3) SIG, (4) Insulin. After 6h of treatment, cells and supernatants were collected for relevant tests, and 3 replicate wells were set for each group.

### Biochemical indicators of mice

Serum samples were collected from mice to determine the serum levels of alanine aminotransferase (ALT), aspartate aminotransferase (AST), cholesterol ester (CHO), triglyceride (TG), high-density lipoprotein (HDL), low-density lipoprotein (LDL), urea nitrogen (UAER), creatinine (CREA), and uric acid (UA) using the automatic biochemical analyzer(Siemens,Germany).

### Enzyme-linked immunosorbent assay

Cell culture supernatants and mouse serum samples, treated with different drugs, were collected for analysis. The levels of IL-6, IL-10, and TNF-α proteins in the supernatants, as well as fasting insulin levels in the serum, were measured following the manufacturer’s instructions for the ELISA kits (Xinbosheng). Absorbance of the cell culture samples was read at 450 nm using a microplate reader, and a standard curve was generated. Each experimental group included three replicates.

### Flow cytometry analysis

Raw264.7 cells, mouse visceral adipose and pancreatic tissues were collected after different drug interventions. Adipose and pancreatic tissues were digested with mixed enzyme solution, filtered through a 200-mesh stainless steel drying net, and red blood cells in cell pellets were lysed. After collecting the cell pellets, Anti-Human/Mouse CD11b PerCP-Cy5.5 and FITC anti-mouse F4/80 were added respectively, and cells were identified after incubation in the dark. The collected Raw264.7 cells and mouse adipose macrophages were washed with pre-cooled PBS, and about 10^6^ cells were added to each tube after cell counting. PE anti-mouse CD86, APC anti-mouse CD163 and appropriate amount of PBS were added, and incubated in the dark for 30 min; the cell pellets were resuspended in PBS, and the samples were filtered through a 300-mesh cell screen for detection. M1 and M2 cells were CD86^+^ and CD163^+^ cells, respectively,(BriCyte E6,Mindray,China).

The mouse serum was diluted, mixed with IL-6, IL-10 and TNF-α beads of Multi-Analyte Flow Assay Kit (Biolegend), shaken and incubated in the dark for 2 h on a shaker at 800 rpm/min. They were washed with a Wash buffer, then incubated with antibodies and in the dark for 1 h. Finally, it was incubated with SA-PE in the dark for 30 min; resuspended in 1× Wash buffer for flow cytometry analyses (FACSLYRIC, BD FACSymphony™, America).

### Oil Red O and HE staining

The abdominal visceral fat, liver, and pancreas tissues of all sacrificed mice were collected, fixed overnight in tissue fixative, incubated in ethanol gradients, and embedded in paraffin. The tissues were cut into 3 mm to 5 mm thickness and stained with Oil Red O, hematoxylin and eosin (HE). Tissue slides were examined imaged using the Nikon optical microscope(E100, Japan).

### Immunohistochemistry

The collected pancreatic tissue sections were dewaxed, incubated with ethanol gradient, washed with PBS twice, immersed in ddH2O containing 3% hydrogen peroxide, incubated at room temperature for 10 minutes to inactivate peroxidase, washed with PBS 3 times, uniformly covered with 3%BSA, closed at room temperature for 30min, washed with PBS twice. CD86 (1:300) and CD163(1:300) primary antibodies diluted with PBS were added and incubated at 4°C overnight. The slices were washed with PBS for 3 times, incubated with secondary antibody at room temperature for 20 min, and soaked in fresh DAB (3,3 ‘-diaminobenzene) solution for 8 min. Brown positive signal on DAB. Tissue slides were examined imaged using the Nikon optical microscope(E100, Japan).

### Western blotting

Protein samples were extracted from Raw264.7 cells and pancreatic tissue of mice treated with different drugs by lysing the specimen with the lysis buffer. The protein concentration was detected by the BCA protein assay kit and mixed with 5×Running buffer at a ratio of 4:1. The sample was boiled at 95°C for 5 min and then separated by SDS-PAGE in Mini PROTEAN electrophoresis tank and transferred to PVDF membrane on the protein transfer instrument (Bio-Rad). The membrane was incubated with 2.5% skim milk powder to block non-specific binding for 2 hours. The membrane was then incubated with GAPDH (1:1000), mTORc1 (1:2000), IKKβ(1:2000),PPAR-γ (1:2000), NF- κB(p-65) (1:5000) and NF-κB(p-p-65) (1:2000) antibodies at 4°C overnight. It was washed with PBS followed by incubation with horseradish peroxidase-labeled goat anti-rabbit secondary antibody (1:10000) at room temperature for 1 hour. After a further wash with PBS, the color was developed using Sunview-ECL ultra-sensitive luminescent liquid in an electrophoresis gel imaging analysis system (Beijing Liuyi Biotechnology Co., Ltd.).

### Transcriptomics analysis of samples

The Raw264.7 cells were incubated with 35 mmol/l hyperglycemia (HG) and 35mmol/l hyperglycemia Sitagliptin phosphate (HG+SIG) for 6 hours (with three replicates per group) and subjected to full transcriptional sequencing. Differentially expressed genes between the two groups were identified based on the (|log_2_ (fold change)|>1) criterion. All bulk RNA-seq data were transformed into TPM and log2, and differential gene analysis was performed using the limma R package v3.54.0 (cite: https://www.ncbi.nlm.nih.gov/pmc/articles/PMC4402510/). Pathway gene set signature was derived from the Molecular Signatures Database (MSigDB) (https://www.gseamsigdb.org/gsea/msigdb) and the ssgsea function from the GSVA R package v1.46.0 as described previously (https://bmcbioinformatics.biomedcentral.com/articles/10.1186/1471-2105-14-7) were utilized to determine the pathway score. The results were visualized using ggplot2 v3.5.0 (https://doi.org/10.32614/CRAN.package.ggplot2).

### Statistical analysis

The data were analyzed using the GraphPad Prism 8 software. The figures were drawn using Adobe Photoshop CS6. Quantitative data were expressed as $\bar{\text{x}}$ ± s, the independent sample T test was used to compare two groups, while one-way analysis of variance was used to compare between multiple groups. P < 0.05 was considered statistically significant.

## Results

### Effects of Sitagliptin phosphate on glucose metabolism and adipose tissue

**Figure 1:**
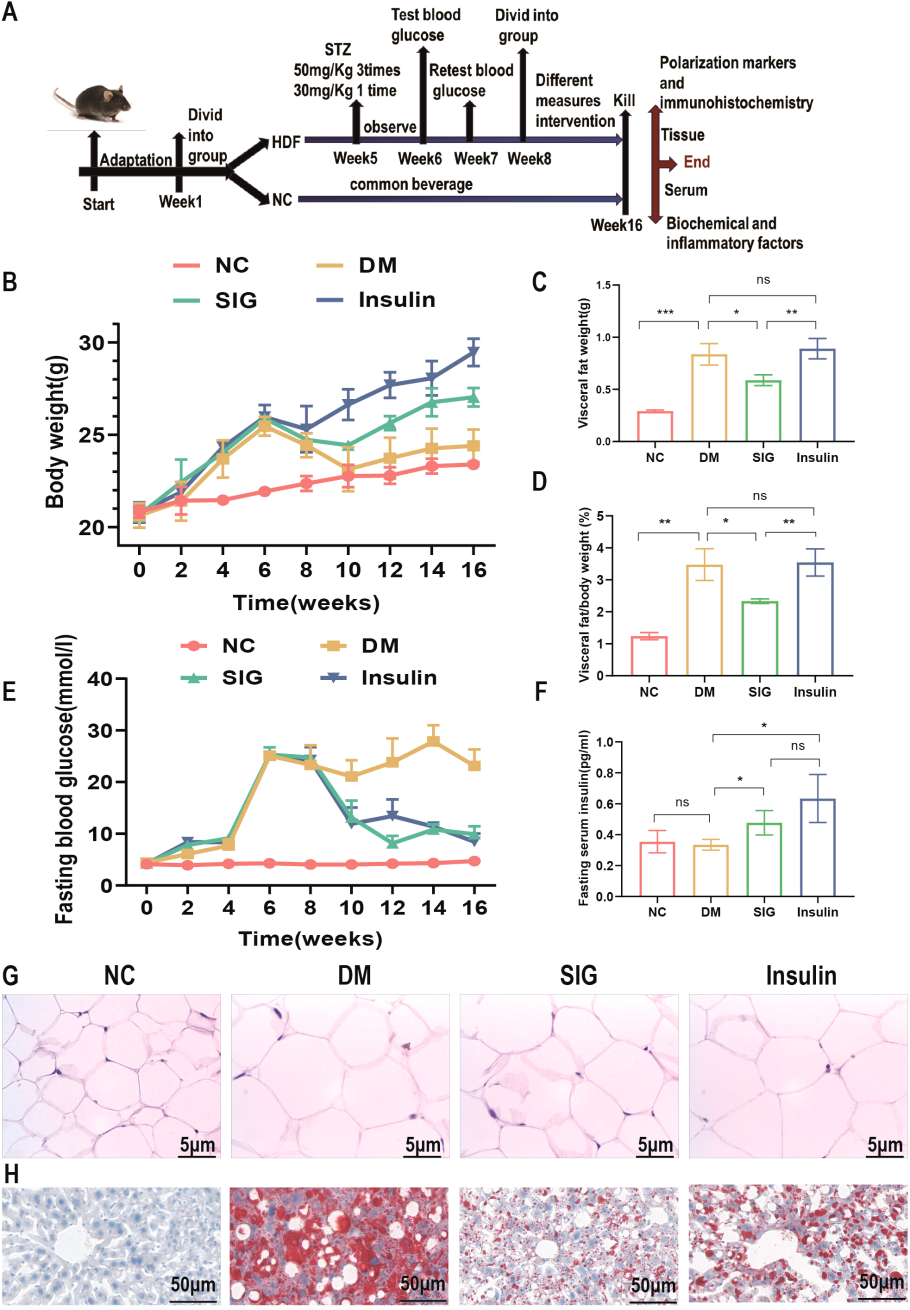
Sitagliptin phosphate reduced the body weight of T2DM mice, reduced the size and weight of adipocytes and the changes in fat/body weight, improved fasting blood glucose and insulin levels, and reduced liver lipid deposition. **(A)** Modeling and drug treatment process of T2DM mice; **(B)** Body weight changes during modeling and treatment; **(C)** Changes in abdominal visceral fat after treatment; **(D)** Changes in abdominal visceral fat/body weight after treatment; **(E)** Changes in fasting blood glucose during modeling and treatment; **(F)** Changes in fasting insulin levels after treatment; Figures for representative sections are shown at ×400 magnification, **(G)** HE staining of abdominal visceral fat cells after drug treatment; **(H)** Liver oil red O staining after drug treatment; *P <0.05, **P <0.01, ***P <0.001; ns, no significance.

To study the effect of Sitagliptin phosphate on glucose metabolism and adipose tissue, HFD+STZ-induced T2DM mice were treated with Sitagliptin phosphate (**Figure 1A**). Compared with the normal group, the body weight (**Figure 1B**) and abdominal visceral fat weight (**Figure 1C**), the ratio of visceral fat weight to body weight (**Figure 1D**), fasting blood glucose (**Figure 1E**) of mice in diabetic group were significant increase, and fasting insulin level did not change much (**Figure 1F**); Sitagliptin phosphate reduced the increase in body weight and abdominal visceral fat weight caused by DM, improved the ratio of visceral fat to body weight and fasting blood glucose, and increased fasting insulin level, and the above indicators were better than those of the insulin group, as shown in **Figures 1B–F**. Compared with the normal group, Histological HE staining showed that DM increased the size of visceral adipocytes (**Figure 1G**), and Oil red O staining showed that DM increased lipid deposition in the liver (**Figure 1H**); compared with the DM group, Sitagliptin phosphate reduced the size of adipocytes and hepatocyte lipid deposition, and the therapeutic effect was significantly better than insulin (Figures GH). The results showed that Sitagliptin phosphate improved glucose metabolism and fat metabolism impairment in T2DM mice. The serum biochemical analysis revealed significantly elevated levels of ALT, AST, CHO, CREA, and UA in diabetic mice compared to the normal group (P < 0.05). Treatment with Sitagliptin phosphate led to a reduction in ALT, AST, and CHO levels, although it did not affect CREA and UA levels. Notably, Sitagliptin phosphate was more effective than insulin Desigulin in reducing AST levels (**Supplementary 1**).

### Effects of Sitagliptin phosphate on inflammatory factors *in vivo* and *in vitro*

**Figure 2:**
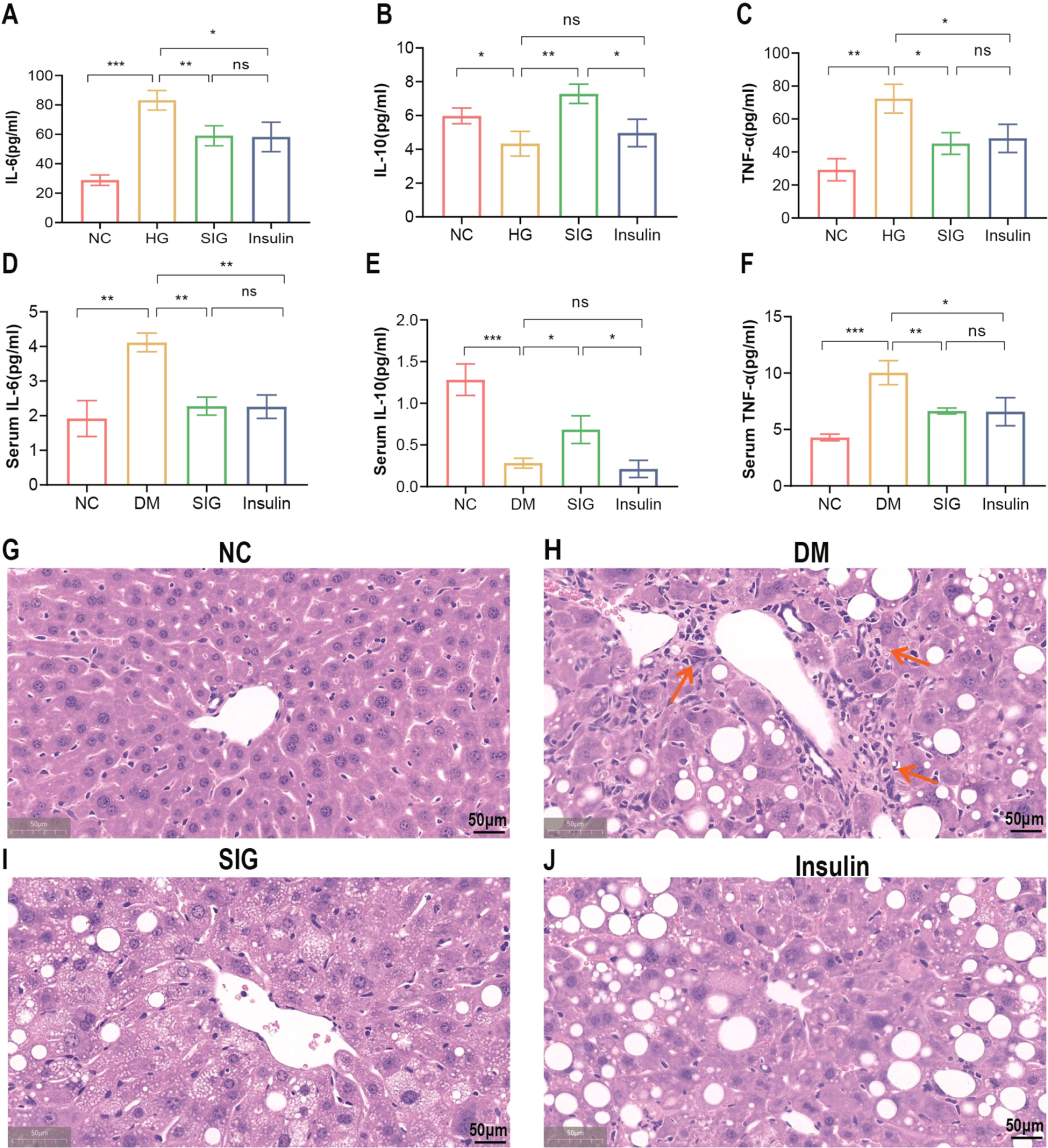
Sitagliptin phosphate reduced the expression of proinflammatory factors in Raw264.7 cells of high glucose intervention and T2DM mice, and inflammatory cell infiltration in the liver of T2DM mice. The levels of IL-6, TNF-α and IL-10 in the supernatant of Raw264.7 cells in each group were treated with different drugs for 6 hours, **(A)** IL-6; **(B)** TNF-α; **(C)** IL-10; Serum levels of IL-6, TNF-α and IL-10 in T2DM mice were treated with different drugs for 8 week, **(D)** IL-6; **(E)** IL-10; **(F)** TNF-α; H&E staining of liver sections was used to analyze the changes of liver lobules, figures for representative sections are shown at ×400 magnification, **(G)** NC; **(H)** DM; **(I)** SIG; **(J)** Insulin. *P < 0.05,**P < 0.01,***P < 0.001; ns, no significance.

To study the effect of Sitagliptin phosphate on inflammation, *in vitro* and *in vivo* experiments were performed. *In vitro* cell experiments confirmed that compared with the normal group, high glucose promoted the expression of inflammatory factors IL-6 and TNF-α secreted by macrophages, and Sitagliptin phosphate and insulin intervention inhibited the expression of IL-6 and TNF-α (**Figures 2A, C**, P <0.05), but there was no statistical difference between the two; compared with the insulin group, Sitagliptin phosphate promoted the expression of anti-inflammatory factor IL-10 (**Figure 2B**, P <0.05). In the *in vivo* experiment, compared with the normal group, the serum levels of IL-6 and TNF-α in the DM group were significantly increased, and Sitagliptin phosphate and insulin intervention inhibited the serum levels of IL-6 and TNF-a (**Figures 2D, F**, P <0.05); compared with insulin intervention, Sitagliptin phosphate improved the decreased expression of IL-10 in DM mice (**Figure 2E**, P <0.05). Histological HE staining of the liver revealed that compared with the normal group (**Figure 2G**), diabetes increased the infiltration of inflammatory factors in the liver portal area (**Figure 2H**), and Sitagliptin phosphate significantly reduced the infiltration of inflammatory factors in the liver portal area (**Figure 2I**), and was superior to the insulin group (**Figure 2J**). *In vivo* and *in vitro* experiments consistently demonstrated that Sitagliptin phosphate inhibited diabetes-induced inflammation by inhibiting proinflammatory factors and increasing the levels of anti-inflammatory factors.

### Effects of Sitagliptin phosphate on macrophage polarization *in vitro* and *in vivo*

**Figure 3:**
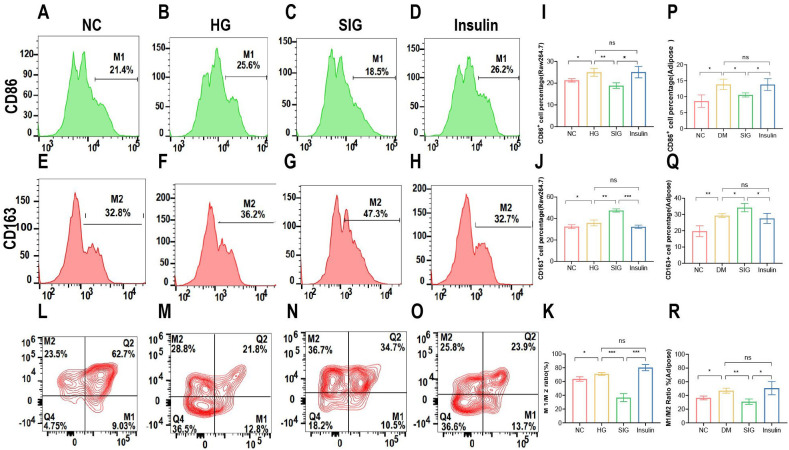
Sitagliptin phosphate reduced the expression percentage of CD86^+^ cells, a M1 polarization marker in Raw264.7 cells treated with high glucose and visceral adipose tissue macrophages in T2DM mice, increased the percentage of CD163^+^ cells, a M2 polarization marker, and reduced the ratio of M1/M2. Raw264.7 cells were treated with different drugs for 6h and collected for flow cytometry, Percentage of CD86^+^ cells in Raw264.7 cells, **(A)** NC; **(B)** HG; **(C)** SIG; **(D)** Insulin; Percentage of CD163 ^+^ cells in Raw264.7 cells, **(E)** NC; **(F)** HG; **(G)** SIG; **(H)** Insulin; **(I)** Comparison of M1 polarization markers; L. **(J)** Comparison of M2 polarization markers; **(K)** M1/M2 ratio. Single cell suspension was prepared by collecting the intra-abdominal visceral adipose tissue of mice with different drug intervention, and then flow cytometry was performed after identification, **(L)** NC; **(M)** DM; **(N)** SIG; **(O)** Insulin; **(P)** Comparison of M1 polarization markers; **(Q)** Comparison of M2 polarization markers; **(R)** M1/M2 ratio. *P < 0.05, **P < 0.01, ***P < 0.001; ns, no significance.

To investigate whether Sitagliptin phosphate reduces inflammation by improving the imbalance of macrophage polarization, *in vitro* and *in vivo* experiments were performed to detect the changes in macrophage subtypes after Sitagliptin phosphate intervention. In the *in vitro* cell experiments, compared with the normal group, high glucose increased the expression of CD86^+^ cells, a polarization marker of M1 macrophages, and CD163^+^ cells, a polarization marker of M2 macrophages, in Raw264.7 cells (**Figures 3B, F**, P < 0.05), Sitagliptin phosphate intervention reduced the expression of CD86^+^ cells (**Figure 3C**, P < 0.05) and increased the expression of CD163^+^ cells (**Figure 3G**, P < 0.05), while insulin intervention had little effect on the changes of CD86^+^ and CD163^+^ cells (**Figures 3D, H**, P > 0.05). In the *in vivo* experiment, compared with the normal group, the expression of CD86^+^ and CD163^+^ cells in the adipose tissue macrophages of T2DM mice increased (**Figure 3M**, P < 0.05), Sitagliptin phosphate decreased the expression of CD86^+^ cells and increased the expression of CD163^+^ cells (**Figure 3N**, P < 0.05), and insulin had little effect on the expression of CD86^+^ and CD163^+^ (**Figure 3O**, P > 0.05). In addition, whether *in vivo* or *in vitro* experiments, Sitagliptin phosphate reduced CD86^+^ (**Figures 3I**, **P**, *P* < 0.05) and increased CD163^+^ cells (**Figures 3J**, **Q**, *P* < 0.05), and more importantly, reduced the ratio of M1/M2 (**Figures 3K, R**, P> 0.05). The results indicated that Sitagliptin phosphate reduced high glucose-induced inflammation by improving the imbalance of macrophage polarization.

### Effects of Sitagliptin phosphate on pancreatic islet tissue in T2DM mice

**Figure 4:**
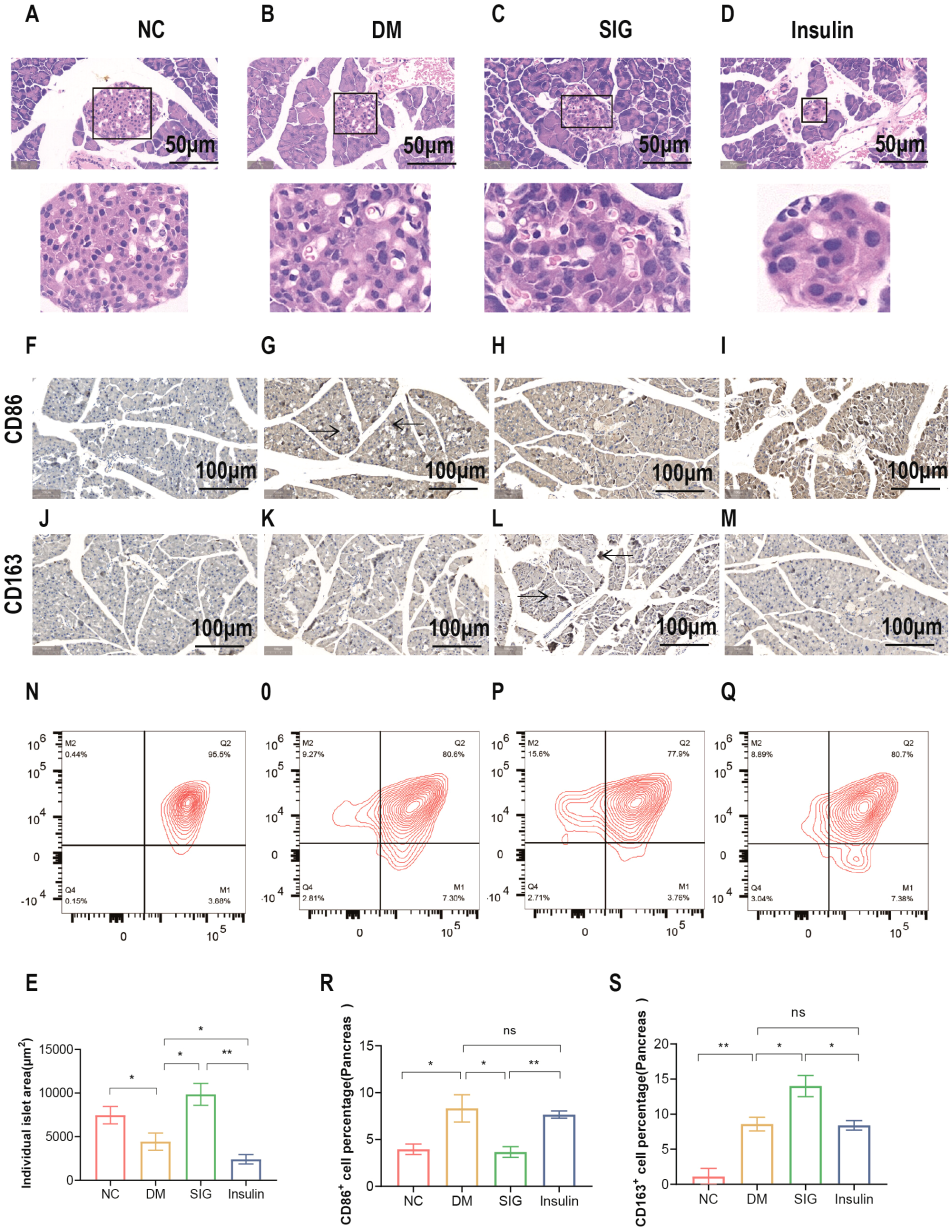
Effects of Sitagliptin phosphate on Pancreatic Islet Morphology and Pancreatic Macrophage Polarization Markers in T2DM Mice. After 8 weeks of treatment with different drugs, pancreatic tissues were collected for HE staining, immunohistochemistry and flow cytometry. Pancreatic HE staining, figures for representative sections are shown at ×400 magnification, **(A)** Normal; **(B)** DM; **(C)** SIG; **(D)** Insulin; **(E)** Comparison of Pancreatic Islet Areas in Mice with Different Interventions; Pancreatic immunohistochemistry, figures for representative sections are shown at ×200 magnification, **(F)** CD86 NC; **(G)** CD86 DM; **(H)** CD86 SIG; **(I)** CD86 Insulin; **(J)** CD 163 NC; **(K)** CD163 DM; **(L)** CD163SIG; **(M)** CD163 Insulin. Pancreatic flow cytometry, **(N)** Normal; **(O)** DM; **(P)** SIG; **(Q)** Insulin, **(R)** Comparison of Pancreatic CD86^+^ Cell Percentages; **(S)** Comparison of Pancreatic CD163^+^ Cell Percentages. *P < 0.05, **P < 0.01, ns, no significance.

To study the effect of Sitagliptin phosphate on pancreatic tissue inflammation and pancreatic macrophage polarization, pancreatic tissue HE staining, immunohistochemical and macrophage flow cytometric analysis were performed. The results of pancreatic histological HE staining showed that in the normal group of mice, islets formed by piles of neuroendocrine cells were observed between the pancreatic lobules. The islets were regular in shape and round or oval, with dark-stained nuclei and abundant cytoplasm (**Figure 4A**); the islets in the T2DM group were irregular in shape, with reduced neuroendocrine cell volume, enlarged nuclei with mild heterogeneity, and reduced cytoplasm (**Figure 4B**); the islets in the Sitagliptin phosphate treatment group were irregular in shape, with enlarged cell volume, and red blood cells were visible in the islets (**Figure 4C**); the cells in the insulin treatment group were reduced in volume, dense nuclei with mild heterogeneity, reduced cytoplasm, and no red blood cells were seen in the islets (**Figure 4D**). The comparison of islet area in mice in different intervention groups is shown in **Figure 4E**. Immunohistochemical analysis of pancreatic tissue revealed an increase in M1 marker CD86+ cells (**Figure 4G**) and M2 marker CD163+ cells (**Figure 4K**) in the DM group compared to the normal group (**Figure 4G**). In contrast, Sitagliptin phosphate treatment reduced the number of CD86+ cells (**Figure 4H**) and increased CD163+ cells (**Figure 4L**) in the DM group. No significant changes were observed in the insulin treatment group (**Figures 4I, M**).Further flow cytometric analysis of pancreatic tissue macrophage polarization markers showed that compared with the normal group, the percentage of M1 marker CD86^+^ cells M2 marker CD163^+^ cells in the pancreatic tissue of mice in the DM group increased (**Figure 4O**, P < 0.05), and Sitagliptin phosphate intervention reduced the percentage of CD86^+^ cells caused by DM and increased the percentage of CD163^+^ cells (**Figure 4P**, P < 0.05), and insulin intervention had little effect on CD86^+^ and CD163^+^ cells (**Figure 4Q**, P > 0.05). Sitagliptin phosphate inhibits the M1-type polarization (**Figure 4R**, *P*<0.05) of pancreatic macrophages and promotes M2-type polarization (**Figure 4S**, *P* <0.05). The results showed that Sitagliptin phosphate alleviated diabetes-induced inflammation and pancreatic islet damage by improving pancreatic islet macrophage polarization.

### Sitagliptin phosphate improves diabetic inflammation and macrophage polarization via mTORc1/PPAR-γ/NF-κB *in vitro* and *in vivo*

**Figure 5:**
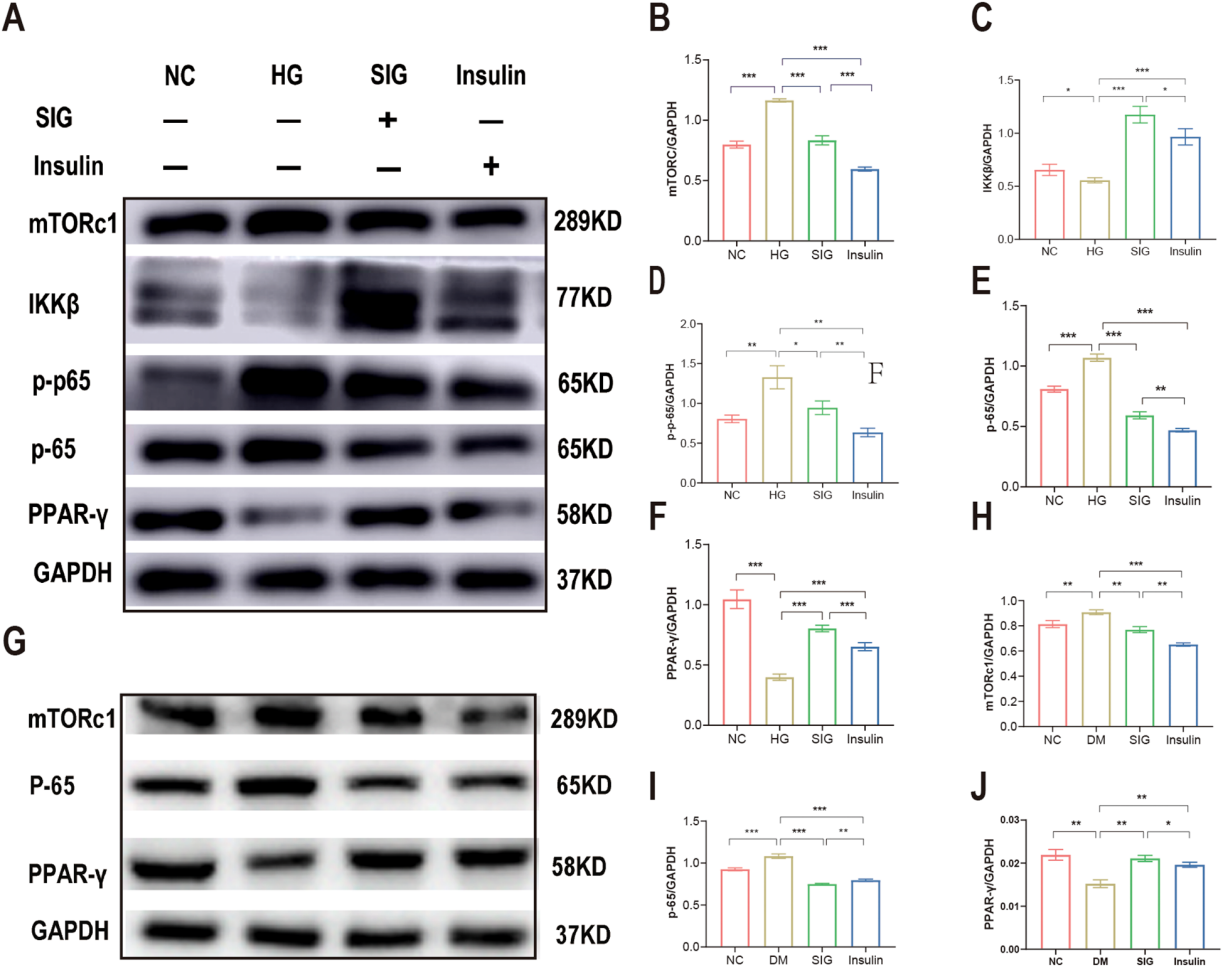
Sitagliptin phosphate improves T2DM inflammation and macrophage polarization via mTORc1/PPAR-γ/NF-κB pathway. Raw264.7 cells were treated with different drugs for 6h, and cells were collected for WB, **(A)** Effects of Sitagliptin phosphate on mTORc1,PPAR-γ and NF-κB in Raw264.7 cells affected by high glucose; **(B)** mTORc1; **(C)** IKKβ; **(D)** PPAR-γ; **(E)** p-65; **(F)** p-p-65;the pancreases of mice treated with different drugs for 8 weeks were collected for WB, **(G)** Effects of Sitagliptin phosphate on mTORc1, PPAR-γ and NF-κB in the pancreas of diabetic mice; **(H)** mTORc1; **(I)** p-65; **(J)** PPAR-γ. *P < 0.05, **P < 0.01, ***P < 0.001.

The mTORc1, PPAR-γ and NF-κB proteins modulate of metabolism and inflammation processes. To investigate the potential pathways targeted by Sitagliptin phosphate in the treatment of diabetic inflammation and macrophage polarization, we measured the expression of mTORc1, PPAR-γ and NF-κB proteins *in vitro* and *in vivo*. Results of WB showed that high glucose stimulation upregulated the expression of mTORc1 protein in Raw264.7 cells (**Figure 5B**, P < 0.05), downregulated the expression of IKKβ (**Figure 5C**, P < 0.05) and PPAR-γ proteins (**Figure 5D**, P < 0.05), promoted P-65 phosphorylation (**Figure 5E**, P < 0.05), and promoted P-65 protein expression compared with the normal group (**Figure 5F**, P < 0.05); Sitagliptin phosphate and insulin intervention reduced the expression level of mTORc1 protein (P < 0.05), increased the expression of IKKβ and PPAR-γ proteins (P < 0.05), and inhibited the expression of p-65 and pp-65 proteins (P < 0.05). Sitagliptin phosphate had a better effect on PPAR-γ protein expression than insulin (P < 0.05), (**Figures 5A–F**). The WB results of animal pancreatic tissue also showed the same results, **Figures 5G–J**.

### Effects of Sitagliptin phosphate on macrophage polarization-related genes based on transcriptomics analysis

**Figure 6:**
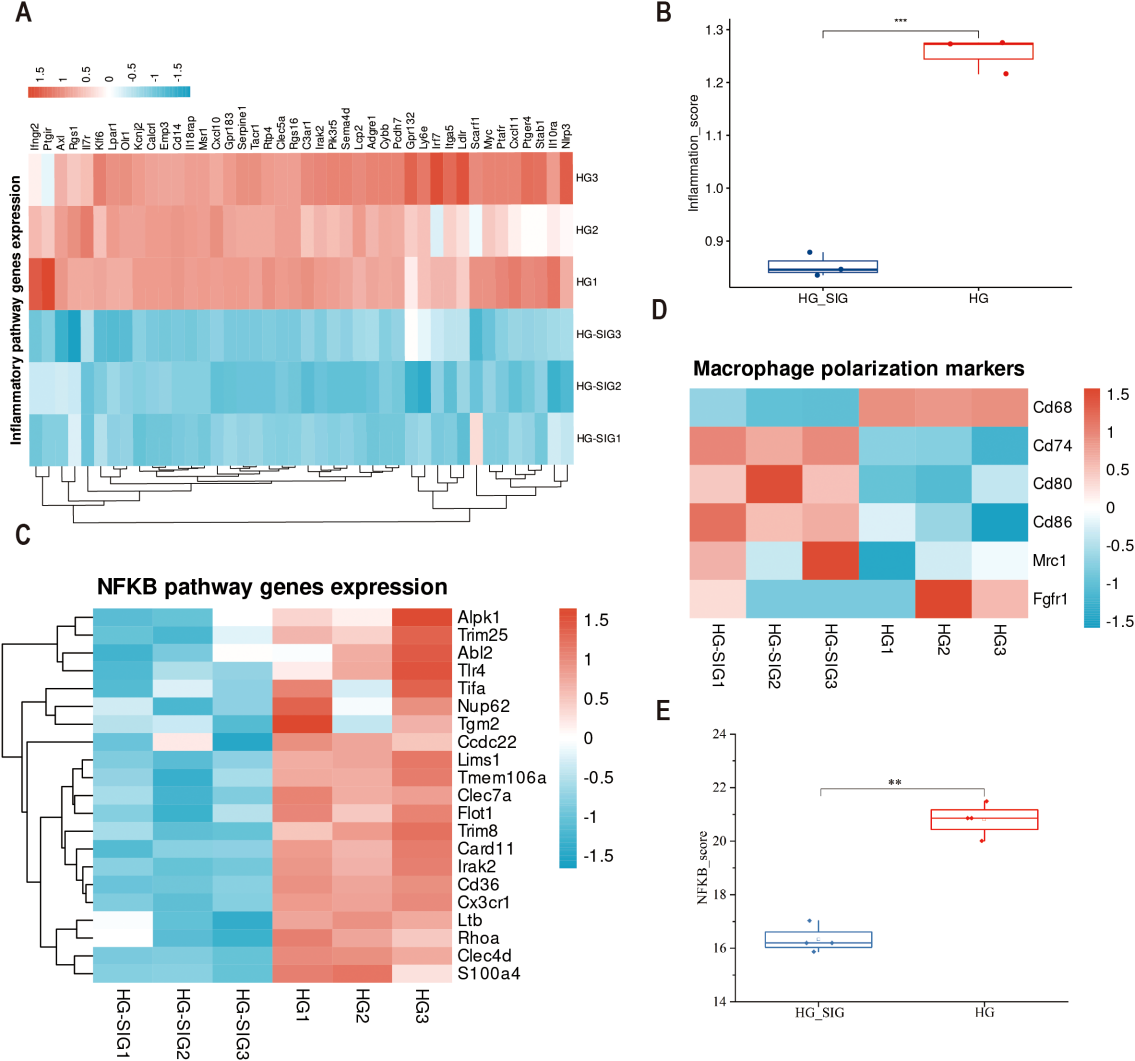
Transcriptomics analysis of gene expression profiles for macrophage. The Raw264.7 cells were incubated with 35 mmol/l hyperglycemia (HG) and 35mmol/l hyperglycemia Sitagliptin phosphate (HG+SIG) for 6 hours (with three replicates per group) and subjected to full transcriptional sequencing. **(A)** A heatmap showing enrichment of inflammatory pathways; **(B)** Comparison of the inflammatory score between HG group and Sitagliptin phosphate treated cells; **(C)** A heatmap showing enrichment of NF-κB pathway enrichment; **(D)** Enrichment of macrophage polarization markers genes. **(E)** Comparison of the inflammatory score between HG group and Sitagliptin phosphate treated cells. **P < 0.01, ***P < 0.001.

Analysis of the transcriptomics data showed that identified 42 inflammation-related genes which were significantly downregulated in cells treated with Sitagliptin phosphate intervention compared with cell in the high-glucose group (**Figures 6 A, B**, P < 0.001). Moreover, clustering analysis of macrophage polarization-related genes among the downregulated genes in inflammatory pathways revealed that M1 polarization-related genes, such as CD68, CD74, CD80, and CD86, were downregulated, while M2 polarization-related genes, such as Mrc1, were upregulated (**Figure 6C**). Gene enrichment analysis for the NF-κB pathway revealed that Sitagliptin phosphate treatment significantly inhibited 21 genes associated with the NF-κB pathway (**Figure 6E**, P < 0.01).

## Discussion

Chronic inflammation in metabolic tissues, such as adipose tissue and the pancreas, contributes to the progression of T2DM. In particular, the accumulation of excess visceral fat is a significant factor driving the occurrence of chronic low-grade inflammation (15). Moreover, cytokines released by visceral adipose tissue enter the bloodstream, affect metabolism, and participate in a variety of metabolic processes (16). Sitagliptin phosphate is a long-acting oral hypoglycemic agent frequently employed in clinical settings. Our study demonstrated that Sitagliptin phosphate not only enhances glucose metabolism in mice with T2DM but also significantly reduces the secretion of pro-inflammatory cytokines. This occurs by inhibiting the polarization of macrophages toward the M1 phenotype while promoting their polarization to the M2 phenotype. Furthermore, Sitagliptin phosphate increases the levels of anti-inflammatory cytokines, thereby effectively mitigating low-grade inflammation in metabolic tissues. Furthermore, we investigated the anti-inflammatory mechanism of Sitagliptin phosphate and discovered that the restoration of macrophage polarization balance following treatment is partially mediated by the activation of PPAR-γ receptors.

In this study, we utilized HFD+STZ-induced T2DM mice and Raw264.7 macrophages exposed to a specific concentration of high glucose as models to explore the effects of Sitagliptin phosphate. The results showed that the Sitagliptin phosphate decreased levels of IL-6 and TNF-α, corroborating the findings reported by Liu et al. (17) and Lin et al. (18). Concerning the effect of Sitagliptin phosphate on IL-10 levels, we found that it significantly enhanced IL-10 expression. This finding aligns with observations by Yu et al. (19), who reported that the administration of adipose tissue-derived mesenchymal stem cells in diabetic rats resulted in a reduction of IL-6 and TNF-α levels, accompanied by the increase in IL-10 levels. High circulating level of IL-6 is considered a hallmark of chronic inflammatory diseases (20). TNF-α is a major regulator of inflammatory diseases (21). Since IL-6 and TNF-α receptors have a common repeating structure, they may promote each other’s expression. Studies have demonstrated that IL-6 regulates glucose metabolism, especially in insulin-sensitive tissues such as pancreatic islets and adipose tissue. Therefore, we speculate that Sitagliptin phosphate may ameliorate glucose metabolism disorders by exerting anti-inflammatory effects (20). The exact anti-inflammatory mechanisms of Sitagliptin phosphate remain to be fully elucidated. However, Rohm et al. (3) suggest that increased macrophage infiltration in metabolic tissues, such as adipose tissue, along with the polarization of macrophages toward the M1 phenotype, contributes to chronic inflammation in diabetes. This study specifically examines the role of macrophage polarization in adipose and pancreatic islet tissues in the context of inflammation in T2DM mice.

Our findings indicate that, both *in vitro* and *in vivo*, Sitagliptin phosphate effectively reduces the polarization of macrophages to the M1 phenotype and lowers the production of pro-inflammatory cytokines. Moreover, it enhances the production of anti-inflammatory cytokines by promoting the polarization of macrophages to the M2 phenotype, thereby effectively managing inflammation. In our *in vitro* experiments, high glucose-stimulated Raw264.7 cells treated with Sitagliptin phosphate showed decreased expression of CD86, a marker associated with M1 polarization, while exhibiting increased expression of CD163, a marker indicative of M2 polarization. In previous animal studies, it was found that Sitagliptin phosphate suppressed the expression of CD86, a marker of macrophage polarization, while simultaneously increasing CD163 expression in the abdominal visceral adipose tissue of T2DM mice. Comparable results were observed in pancreatic islet tissue. Notably, treatment with Sitagliptin phosphate significantly reduced the M1/M2 ratio in both *in vitro* and *in vivo* experiments, indicating a shift toward a more anti-inflammatory macrophage profile. Chawla et al. (22) suggested that modulating the M1/M2 ratio of macrophages is a more effective strategy for controlling inflammation than merely altering the total number of macrophages. Similarly, Yu et al. (19) proposed that a decrease in the M1/M2 ratio may indicate a systemic reduction in inflammation rather than a localized effect in a specific target organ. This anti-inflammatory response appears to involve a phenotypic shift from M1 macrophages to M2 macrophages.

**Figure 7:**
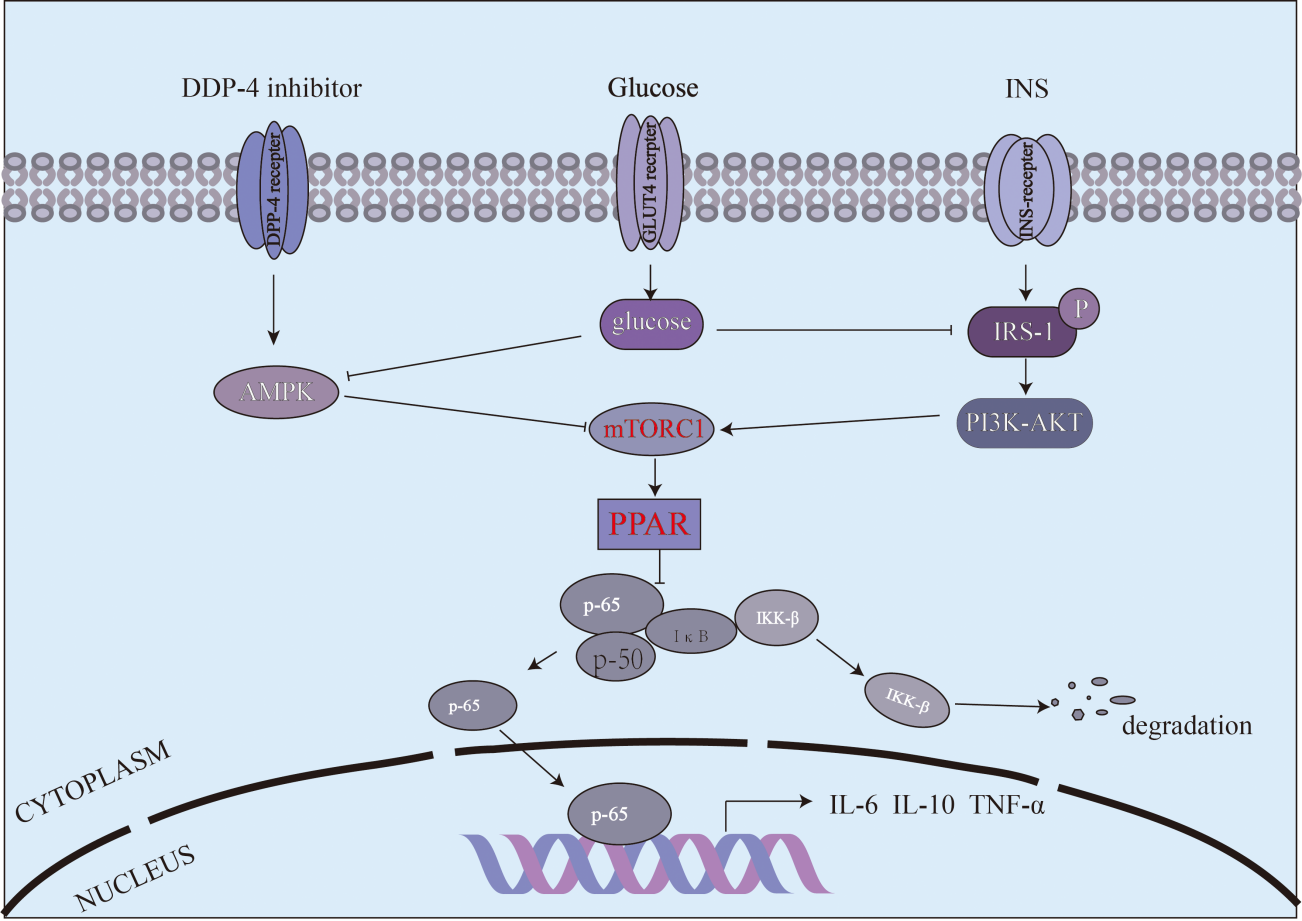
The mechanism by which Sitagliptin phosphate improves high-glucose induced macrophage polarization and inflammation via the mTORc1/PPAR-γ/NF-κB pathway.

Mammalian Target of Rapamycin Complex 1 (mTORc1) is involved in the regulation of energy balance and metabolism, and is also an important part of the signaling pathway activated by inflammatory mediators (23). PPAR-γ,a downstream protein of mTORC1,is a ligand-activated transcription factor belonging to the peroxisome proliferator-activated receptor (PPAR) nuclear receptor family. Its main function is to regulate the expression of genes involved in lipid and glucose metabolism, while also promoting the differentiation of adipocytes (24). Chawla et al. (25) proposed that PPAR-γ activation diminishes the secretion of pro-inflammatory factors by macrophages, indicating its negative regulatory role in classical macrophage activation. Elsewhere, Odegaard et al. (26) demonstrated that mice lacking macrophage-specific PPAR-γ exhibited a 20% increase in body weight and total fat mass, underscoring the importance of PPAR-γ in the maturation of activated macrophages. In this study, *in vitro* WB results indicated that Sitagliptin phosphate ameliorated the reduced expression of PPAR-γ in Raw264.7 macrophages induced by high glucose levels. Moreover, WB analysis of pancreatic tissue from T2DM mice demonstrated that PPAR-γ expression was decreased, but treatment with Sitagliptin phosphate upregulated its expression. Moreover, the increase of PPAR-γ expression inhibited NF-κB expression and reduced the secretion of inflammatory cytokines such as IL-6 and TNF-α. Moreover, increased PPAR-γ levels enhanced the secretion of the anti-inflammatory cytokine IL-10, which may further promote the polarization of macrophages toward the M2 phenotype. Bouzazi et al. (27) proposed that IL-10 may the key factor driving macrophage polarization to M2. Accumulating evidence has shown that IL-10 is the main inflammatory factor regulating macrophage polarization and persistent IL-10 signaling maintains this phenotype (28). The proposed mechanism is shown in **Figure 7**. To validate the effect of high-glucose and Sitagliptin phosphate treatment on the expression of inflammation-related genes and macrophage polarization, we conducted transcriptomics analysis on Raw264.7 cells exposed to high glucose and Sitagliptin phosphate. This analysis showed that the Sitagliptin phosphate abolished the increase in 42 inflammation-related genes, which were enhanced by high-glucose treatment. Moreover, gene enrichment analysis predicted that the NF-κB pathway could mediate the inhibitory effects of Sitagliptin phosphate treatment on 21 genes related to the NF-κB pathway.

## Conclusion

In conclusion, this study demonstrates that Sitagliptin phosphate exerts a significant anti-inflammatory effect on type 2 diabetic mice and high-glucose-stimulated Raw264.7 macrophages, effectively mitigating chronic inflammation associated with diabetes. The anti-inflammatory effects of Sitagliptin phosphate are associated with a shift in macrophage polarization, leading to a decrease in the M1 phenotype and an increase in the M2 phenotype. IL-10 plays a crucial role in this M2 polarization, partly by inhibiting NF-κB through PPAR-γ activation and suppressing mTORC1 signaling. Our study has several limitations. First, there are inherent differences between the physiological systems of animals and humans, and the T2DM mouse model may not completely replicate the conditions observed in T2DM patients. Additionally, pharmacological effects *in vivo* can vary due to genetic differences. In future clinical studies, we aim to compare the effects of sitagliptin phosphate on blood glucose levels in T2DM patients with varying levels of inflammation. This will help establish a foundation for regulating macrophage polarization as a strategy to improve chronic inflammation in the treatment of T2DM.

## Data Availability

The original contributions presented in this study are publicly available in the GEO database. The dataset can be accessed here: NCBI GEO Dataset GSE293313. This dataset includes gene expression data relevant to the study and is available for further analysis and validation.
