# Supplementary material for: Sitagliptin phosphate ameliorates chronic inflammation in diabetes mellitus via modulating macrophage polarization
Source: Front Endocrinol (Lausanne). 2025 Apr 7;16:1544684. doi: 10.3389/fendo.2025.1544684 (PMC12010097; doi:10.3389/fendo.2025.1544684)
Supplement: Supplementary file 1 [file Table1.docx]

Note: Different letters in the same column indicate significant differences（*P*<0.05）；the same letter in the same column means the difference is not significant（*P*>0.05）；
